# Sex differences in the genetics of sarcoidosis across European and African ancestry populations

**DOI:** 10.3389/fmed.2023.1132799

**Published:** 2023-05-11

**Authors:** Ying Xiong, Susanna Kullberg, Lori Garman, Nathan Pezant, David Ellinghaus, Vasiliki Vasila, Anders Eklund, Benjamin A. Rybicki, Michael C. Iannuzzi, Stefan Schreiber, Joachim Müller-Quernheim, Courtney G. Montgomery, Johan Grunewald, Leonid Padyukov, Natalia V. Rivera

**Affiliations:** ^1^Respiratory Medicine Division, Department of Medicine Solna, Karolinska Institutet, Stockholm, Sweden; ^2^Department of Respiratory Medicine and Allergy, Theme Inflammation and Ageing, Karolinska University Hospital, Stockholm, Sweden; ^3^Genes and Human Disease, Oklahoma Medical Research Foundation, Oklahoma City, OK, United States; ^4^Institute of Clinical Molecular Biology, Christian-Albrechts-University of Kiel, Kiel, Germany; ^5^Department of Public Health Sciences, Henry Ford Health System, Detroit, MI, United States; ^6^Zucker School of Medicine, Staten Island University Hospital, Northwell/Hofstra University, Staten Island, NY, United States; ^7^Clinic for Internal Medicine I, University Hospital Schleswig-Holstein, Kiel, Germany; ^8^Department of Pneumology, University Medical Center Freiburg, Faculty of Medicine, University of Freiburg, Freiburg im Breisgau, Germany; ^9^Center for Molecular Medicine, Karolinska Institutet, Stockholm, Sweden; ^10^Division of Rheumatology, Department of Medicine Solna, Karolinska Institutet, Karolinska University Hospital, Stockholm, Sweden

**Keywords:** sarcoidosis, genetics, genome-wide association study (GWAS), meta-analysis, single nucleotide polymorphisms, immunogenetics and HLA

## Abstract

**Background:**

Sex differences in the susceptibility of sarcoidosis are unknown. The study aims to identify sex-dependent genetic variations in two clinical sarcoidosis phenotypes: Löfgren’s syndrome (LS) and non-Löfgren’s syndrome (non-LS).

**Methods:**

A meta-analysis of genome-wide association studies was conducted on Europeans and African Americans, totaling 10,103 individuals from three population-based cohorts, Sweden (*n* = 3,843), Germany (*n* = 3,342), and the United States (*n* = 2,918), followed by an SNP lookup in the UK Biobank (UKB, *n* = 387,945). A genome-wide association study based on Immunochip data consisting of 141,000 single nucleotide polymorphisms (SNPs) was conducted in the sex groups. The association test was based on logistic regression using the additive model in LS and non-LS sex groups independently. Additionally, gene-based analysis, gene expression, expression quantitative trait loci (eQTL) mapping, and pathway analysis were performed to discover functionally relevant mechanisms related to sarcoidosis and biological sex.

**Results:**

We identified sex-dependent genetic variations in LS and non-LS sex groups. Genetic findings in LS sex groups were explicitly located in the extended Major Histocompatibility Complex (xMHC). In non-LS, genetic differences in the sex groups were primarily located in the MHC class II subregion and *ANXA11*. Gene-based analysis and eQTL enrichment revealed distinct sex-specific gene expression patterns in various tissues and immune cell types. In LS sex groups, a pathway map related to antigen presentation machinery by IFN-gamma. In non-LS, pathway maps related to immune response lectin-induced complement pathway in males and related to maturation and migration of dendritic cells in skin sensitization in females were identified.

**Conclusion:**

Our findings provide new evidence for a sex bias underlying sarcoidosis genetic architecture, particularly in clinical phenotypes LS and non-LS. Biological sex likely plays a role in disease mechanisms in sarcoidosis.

## Background

Sarcoidosis is a multi-system disease with unknown etiology, characterized by non-caseating granulomas (1). In 90% of cases, sarcoidosis affects the lungs and lymphatic system (2).

Sarcoidosis is prevalent worldwide, commonly affecting individuals between 20 and 50 years of age, with males diagnosed earlier than females (3, 4). On a global scale, sarcoidosis incidence and prevalence vary based on sex, age, geography, and ethnicity (4). In the United States, the prevalence is 141 per 100,000 in African–Americans and 49.8 per 100,000 in Caucasians (5). In Nordic countries such as Sweden, the prevalence of sarcoidosis was reported between 152 and 215 per 100,000 (6), whereas in Denmark was reported at 77 per 100,000 (7).

The disease is heterogeneous, as different manifestations and clinical outcomes have been observed in clinical settings, particularly among sex groups (3, 8, 9). Interestingly, sarcoidosis is more common in females, has a late-onset, has a variable disease course depending on the affected organ, and has a higher mortality rate than men (3). Sex differences are also seen in extra-pulmonary phenotypes (10, 11), including cardiac (12, 13) and skin sarcoidosis.

Sex hormones have been shown to play a role in sarcoidosis (14, 15). For instance, sarcoidosis has a low disease activity in pregnancy or goes into remission. However, the ameliorating effect is lost as flares occur after delivery (16, 17).

In the lungs, sarcoidosis is characterized by Löfgren’s syndrome (LS) and non-Löfgren’s syndrome (LS). LS is an acute form of the disease characterized by periarticular swelling around the ankles, erythema nodosum, and bilateral hilar lymphadenopathy (18). Typically, females with LS exhibit erythema nodosum (EN), while males with LS have symptoms of bilateral ankle arthritis. Patients with LS usually have a good prognosis (19). LS often occurs in individuals of European ancestry and is relatively rare in individuals of African origin. In non-LS, sarcoidosis is characterized by a heterogeneous disease course, often with an insidious onset, unrelenting disease course, and a high risk for clinical organ impairment, predominantly pulmonary fibrosis (18, 20). Patients with fibrotic sarcoidosis have markedly decreased survival compared with the general population (21, 22).

The causes for clinical differences and disease course in sarcoidosis among sex groups are unknown. However, evidence-based factors, including genetics, epigenetics, and environmental exposures, have been postulated. Regarding genetics, few studies have addressed sex differences in the genetic variation in disease, despite most studies adjusting for sex in their analyses (23). An exception is a genome-wide admixture scan conducted in African Americans stratified by sex, identifying several genetic variants associated with sarcoidosis exclusively in females (24).

In this work, we sought to investigate the genetic associations in the sex groups and identify differences and commonalities in European and African population ancestries by a meta-analysis approach and high-density mapping.

## Materials and methods

### Samples and study design

The samples were from three independent sarcoidosis population-based cohorts from Sweden, Germany, and the United States. All participants provided written informed consent for the study. Study protocols of all studies had been approved by respective local institutional boards.

#### The Swedish cohort

The case-control study consisted of 4,133 individuals. The study was approved by the local institutional review board in Stockholm, Sweden. All participants provided written informed consent and were permitted to use their DNA for research purposes. Baseline clinical and demographic characteristics were measured when the participants were enrolled in the study.

Patients were enrolled at the time of disease investigation at the Sarcoidosis Centrum, Karolinska University Hospital Solna, Sweden. The diagnosis was established on radiographic manifestations, findings at bronchoscopy with bronchoalveolar lavage (BAL), including an elevated CD4/CD8 ratio > 3.5, and positive biopsies and in accordance with the criteria outlined by the World Association of Sarcoidosis and Other Granulomatous Disorders [WASOG, Statement on sarcoidosis (27)]. Sarcoidosis patients were further characterized into two clinical phenotypes, LS and non-LS. LS was defined by typical clinical manifestations with an acute onset of the disease, including fever, bilateral hilar lymphadenopathy on chest X-ray, bilateral ankle arthritis, and/or erythema nodosum. Non-LS was defined as a heterogeneous disease course with an insidious onset, unrelenting disease course, and a high risk for clinical organ impairment, such as developing fibrosis in the lung. Healthy controls included 3,085 individuals who were recruited from two large-scale epidemiological cohorts. Mainly, 2,025 individuals were from the Environmental Investigation of Rheumatoid arthritis (EIRA) study described in Klareskog et al. (25) and 1,060 individuals were from the Epidemiological investigation of risk factors for Multiple Sclerosis (EIMS) study described in Hedstrom et al. (26).

#### The German cohort

The case-control study consisted of 4,975 individuals, of which 413 were non-LS (27). The description and inclusion of these patients are described elsewhere (28, 29). Briefly, German control subjects (*n* = 4,498) were derived from Popgen (*n* = 2,485) (30), and the Heinz Nixdorf RECALL (HNR) study (*n* = 1,499) (31). Additionally, 304 control individuals of South German origin were recruited from the Bavarian Red Cross, and 210 control individuals were recruited from the Charité - Universitätsmedizin Berlin. The mean age and male percentage were 62.5 years (SD = 12.3) and 40% male in non-LS and 57.8 years (SD = 12.2), and 51% male in controls.

#### The USA African American cohort

The case-control study consisted of 1,657 individuals. The sample included 781 sarcoidosis cases characterized as non-LS and 876 healthy controls. All individuals were taken from an extensive cohort of African-American (AA) sarcoidosis patients and controls assembled from various studies. Further details are available in Refs. (32–35).

### Genotyping and quality control

Genotyping for sarcoidosis patients in the Swedish cohort was performed using the Illumina Immunochip platform and was performed at The SNP&SEQ Technology Platform in Uppsala University, Sweden, with Illumina Infinium assay using the Immuno Bead Chip (Immunochip version 1) as described in Rivera et al. (23). Healthy controls in the EIRA cohort (*n* = 2,086) were genotyped on the same platform at the Genome Institute of Singapore, as described previously (36, 37). Healthy controls in the EIMS cohort (*n* = 1,060) were genotyped with the same SNP array described elsewhere (26). Briefly, quality control filtering thresholds were applied using tools implemented in PLINK v1.09b software (38, 39). SNPs at call rate < 95% with minor allele frequency (MAF) > 1% and call rate < 99% with MAF ≤ 5% were filtered out. Moreover, SNPs that had Hardy–Weinberg Equilibrium (HWE) *P* < 1 × 10^–7^ (in the control group) were also excluded. Individuals with missing genotypes < 97% were also removed. Quality control (QC) resulted in 141,151 SNPs and 3,842 individuals on the Illumina Immunochip array.

Genotypes for the German cohort were quality controlled, as described in Fischer et al. (40). Genotyping for the African American cohort (US-AA) was performed at the OMRF using the Illumina HumanOmni1-Quad array for ∼1.1M variants across the genome. Of these, 121,988 were in common with the Immunochip platform and carried forward for analysis. Further details on genotyping and quality control filtering are described in Adrianto et al. (41).

### Statistical analysis

#### Association analysis of LS and non-LS in sex groups

In each sex group, we examined the association of single nucleotide polymorphisms (SNPs) in LS and non-LS. For assessing the association in LS, we applied an additive model using logistic regression adjusted for age and four principal components (PCs). PCs were derived from principal component analysis (PCA) performed using EIGENSTRAT (42) software on a pruned genotyped data set. The pruning of genotypes was performed using the pruning function and default parameters implemented in PLINK 1.90 beta software (39). For assessing the association in non-LS, we applied an additive model using logistic regression without covariate adjustment due to the lack of age information across the cohorts. In LS and non-LS, a significance threshold was defined by genomic control-corrected *P* < 3.5 × 10^–7^ (0.05/141,151 quality-controlled SNPs) considering Bonferroni correction. A suggestive *P* < 5 × 10^–5^ was also considered to identify potential signals (43–46). Furthermore, to account for high linkage disequilibrium (LD) (*r*^2^ ≥ 0.8) among associated SNPs, LD pruning was performed using PriorityPruner version 0.01.4 software^1^ with default parameters, which identified and prioritized SNPs in genomic loci and thus referred as tag-SNPs.

As an exploratory analysis, to assess the effect of sex on genetics, we implemented an interaction model where the disease outcome was regressed on SNP adjusted for sex and the interaction term (SNP × sex) as covariates. A significant threshold for the interaction term was set at *P* < 0.05. The interaction analysis was performed using the glm function implemented in PLINK 2.0 software (39).

Additionally, we also performed a heterogeneity test to assess the effects of association between males and females. Heterogeneity statistics i.e., Cochran’s Q-statistic, *I*^2^ statistic (the percentage of variability in the effect sizes), and tau^2^ (the between-study variance in our meta-analysis) were computed applying a random effects model and using the rma function implemented in the metafor R package (47).

#### Meta-analysis of significant SNPs

Meta-analysis was performed using SNP association results in non-LS. Herein, the meta-analysis was conducted using the inverse variance weighting (IVW) method implemented in the METAL software (48). For each SNP, the combined genetic effect size (defined by Beta), standard error (SE), meta-*P*-value (*P*_*meta*_), total variability in effect size, also known as the heterogeneity index (*I*^2^), and heterogeneity *P*-value were calculated. SNPs at *P*_*meta*_ < 5 × 10^–8^ were defined as genome-wide significant. Two meta-analyses were conducted, (1) a meta-analysis considering SNP associations in the European ancestry cohorts (Sweden and Germany) and (2) a meta-analysis considering SNP associations in the multi-ethnic (Sweden, Germany, and US African American).

Besides METAL computing heterogeneity index (*I*^2^), we also performed an independent heterogeneity test applying the random effects model using the rma function implemented in the metafor R package (47). Summary of heterogeneity statistics include Cochran’s Q-statistic, *I*^2^ statistic (the percentage of variability in the effect sizes), and tau^2^ (the between-study variance in our meta-analysis).

#### Lookup of significant SNPs in the UK Biobank

Using the UKB resource, we examined SNP associations at *P* < 5 × 10^–5^ using summary statistics of a genome-wide association study (GWAS) on “doctor-diagnosed sarcoidosis” (Data-Field ID: 22133), consisting of 91,787 individuals (395 cases and 91,392 controls) in the UKBB (49). Chiefly, sex-stratified summary statistics GWAS on males (189 cases and 41,045 controls) and females (206 cases and 50,347 controls) were obtained from https://github.com/Nealelab/UK_Biobank_GWAS. GWAS analysis was conducted using an additive model and logistic regression adjusted by the first 20 PCs, age, and age^2^. A lookup significance threshold was set at *P* < 5 × 10^–8^.

### Gene-based analysis

A gene-based analysis was conducted using SNP associations at *P* < 5 × 10^–5^ and MAGMA (50). Gene-based significance was set to *P* < 2.0 × 10^–6^ based on Bonferroni correction (0.05/24,769 genes). The number of genes was adopted from the VEGAS2 software definition, where 24,769 unique genes on the 22 autosomes were identified (51).

#### SNP enrichment analysis via expression quantitative trait loci (eQTLs) and pathway analysis

Expression quantitative trait loci enrichment was performed using SNP associations at *P* < 5 × 10^–5^ and gene expression data from 53 human tissues from the Genotype-Tissue Expression (GTEx) project version 8 available on the Functional Mapping and Annotation of genetic associations (FUMA) web tool (52). Additional eQTL enrichment using gene expression of lung tissue, whole blood, and immune cell types was conducted using EUGENE software (53, 54). A significant threshold for n SNP correlated with gene expression (defined as eQTL-SNP or eSNP) was set at *P* < 0.05.

Pathway analysis was conducted based on SNP associations at *P* < 5 × 10^–8^ to elucidate disease mechanisms using MetaCore*™* software.

## Results

The summary of phenotypes and the number of samples in the sex groups for cohorts investigated are shown in Table 1.

**TABLE 1:** Sample size in association analysis in Sweden, Germany, and United States (A); sample size in replication lookup in the UK Biobank (B).

(A)				
Cohorts	LS		Non-LS	
	Males	Females	Males	Females
Swedish	166 cases vs. 849 HC	132 cases vs. 2,236 HC	263 cases vs. 855 HC	181 cases vs. 2,245 HC
German			470 cases vs. 1,750 HC	732 cases vs. 1,692 HC
USA-AA			322 cases vs. 497 HC	951 cases vs. 1,148 HC
**(B)**				
**Males**			**Females**	
344 cases vs. 166,644 controls			361 cases and 193,792 controls	

HC, healthy controls.

### Association results

The genome-wide association analysis using high-density mapping Immunochip array was conducted in males and females independently in Swedish, German, and US-African American cohorts.

#### LS sex groups

**FIGURE 1:**
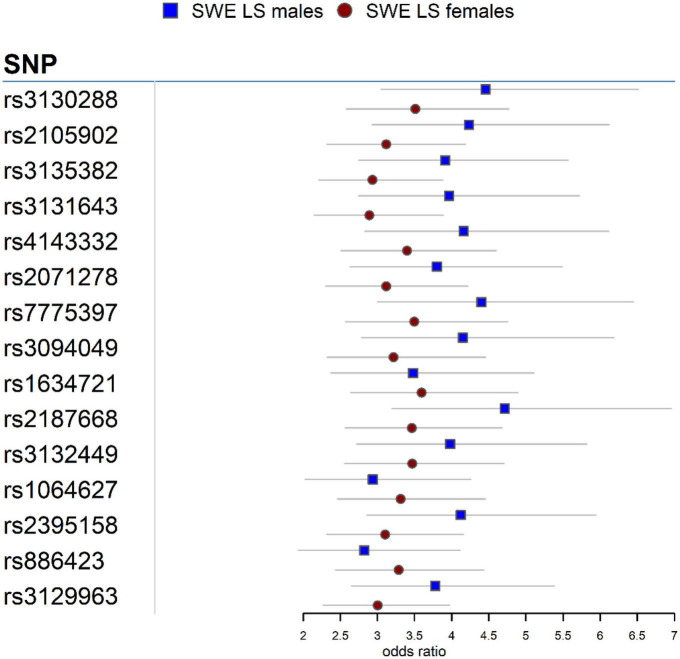
Forest plot for the 15 top common SNPs in the LS sex groups in the Swedish cohort.

In the Swedish cohort (Supplementary Tables 1, 2), 542 SNPs in LS males and 617 SNPs in LS females were identified at *P* < 3.5 × 10^–7^. Rs2187668 (OR = 4.715, SE = 0.1983, *P* = 6.02 × 10^–14^) located in *HLA-DQA1* was the top signal identified in males, and rs9268219 (OR = 3.667, SE = 0.1586, *P* = 3.33 × 10^–15^) located in *C6orf10* was the top signal identified in females. After considering linkage disequilibrium (LD) among associated signals, 77 SNPs in males and 101 SNPs in females remained, which were defined as tag-SNPs. The top results for LS sex groups (Table 2) are illustrated in the forest plot (Figure 1). Manhattan and QQ plots are shown in Supplementary Figure 1. Heterogeneity statistics are available in Supplementary Table 2X.

**TABLE 2:** The top tag-SNPs (P_*GC*_ < 3.5 × 10^–7^) associated with LS males and females in the Swedish cohort.

SNP	CHR	BP (hg19)	Alleles	RefSeq genes	Sex group	CA	CAF	OR	95% CI	*P*_*GC*_
rs2187668	6	32,605,884	A/G	*HLA-DQA1*	LS-M	A	0.3557	4.7150	(3.1966, 6.9547)	6.02E-14
					LS-F	A	0.3557	3.4630	(2.5643, 4.6768)	6.57E-15
rs3130288	6	32,096,001	A/C	*ATF6B*	LS-M	A	0.3456	4.4560	(3.0489, 6.5124)	1.29E-13
					LS-F		0.3456	3.5090	(2.58, 4.7725)	1.39E-14
rs2105902	6	32,395,698	A/T	*12kb 5′ of HLA-DRA*	LS-M	A	0.4010	4.2330	(2.9272, 6.1214)	1.87E-13
					LS-F		0.4010	3.1160	(2.32, 4.1851)	3.76E-13
rs3135382	6	32,383,441	C/A	*8.5kb 5′ of BTNL2*	LS-M	C	0.4446	3.9120	(2.749, 5.567)	3.47E-13
					LS-F		0.4460	2.9310	(2.2128, 3.8823)	5.50E-13
rs3131643	6	31,442,782	A/G	*2.6kb 3′ of HCG26*	LS-M	A	0.3788	3.9640	(2.7471, 5.7201)	1.59E-12
					LS-F		0.3788	2.8890	(2.1476, 3.8864)	1.57E-11
rs4143332	6	31,348,365	A/G	*19kb 5′ of MICA*	LS-M	A	0.3389	4.1600	(2.8303, 6.1145)	3.32E-12
					LS-F		0.3389	3.3970	(2.5095, 4.5985)	2.66E-14
rs2071278	6	32,165,444	G/A	*NOTCH4*	LS-M	G	0.3725	3.8000	(2.6308, 5.4888)	8.55E-12
					LS-F		0.3725	3.1160	(2.3032, 4.2156)	1.36E-12
rs3094049	6	30,359,360	A/G	*45kb 3′ of RPP21*	LS-M	A	0.2903	4.1510	(2.7873, 6.1819)	1.78E-11
					LS-F		0.2903	3.2150	(2.3198, 4.4557)	1.53E-11
rs2524069	6	31,244,789	T/A	*4.9kb 5′ of HLA-C*	LS-M	T	0.3540	3.6090	(2.5168, 5.1752)	2.11E-11
					LS-F		0.3540	2.7470	(2.0481, 3.6845)	8.72E-11
rs2736157	6	31,600,820	G/A	*PRRC2A*	LS-M	G	0.4161	3.4380	(2.4292, 4.8657)	2.22E-11
					LS-F		0.4161	2.7580	(2.0587, 3.6949)	6.19E-11
rs9268219	6	32,284,108	C/A	*C6orf10*	LS-M	C	0.3356	4.4090	(3.0003, 6.4792)	4.10E-13
					LS-F		0.3356	3.6670	(2.6872, 5.004)	3.33E-15
rs1634721	6	30,977,680	A/G	*PBMUCL1*	LS-M	A	0.3221	3.4810	(2.372, 5.1085)	9.45E-10
					LS-F		0.3221	3.5930	(2.6382, 4.8934)	6.08E-15
rs3132449	6	31,626,013	A/G	*25bp 3′ of APOM*	LS-M	A	0.3418	3.9780	(2.7203, 5.8173)	8.08E-12
					LS-F		0.3418	3.4680	(2.5569, 4.7038)	1.47E-14
rs1064627	6	30,698,541	G/A	*FLOT1*	LS-M	G	0.354	2.9360	(2.0263, 4.2541)	4.61E-08
					LS-F		0.354	3.3110	(2.4618, 4.4532)	2.64E-14
rs9266669	6	31,348,077	A/G	*19kb 5′ of MICA*	LS-M	A	0.3625	3.3530	(2.3232, 4.8393)	5.56E-10
					LS-F		0.3625	3.3570	(2.4872, 4.531)	2.73E-14
rs886423	6	30,782,205	G/C	*70kb 5′ of DDR1*	LS-M	G	0.3322	2.8220	(1.9374, 4.1107)	2.11E-07
					LS-F		0.3322	3.2850	(2.4358, 4.4303)	6.69E-14
rs3129963	6	32,380,208	G/A	*5.3kb 5′ of BTNL2*	LS-M	G	0.4513	3.7770	(2.6521, 5.3791)	1.53E-12
					LS-F		0.4513	3.0010	(2.2666, 3.9734)	1.61E-13
rs2233974	6	31,080,016	C/G	*C6orf15*	LS-M	C	0.3793	3.3470	(2.325, 4.8184)	4.47E-10
					LS-F		0.3793	3.1680	(2.3592, 4.2542)	1.66E-13
rs3130477	6	31,428,920	G/A	*2kb 5′ of HCP5*	LS-M	G	0.3389	4.0890	(2.7858, 6.0019)	5.00E-12
					LS-F		0.3389	3.2220	(2.3816, 4.359)	2.91E-13

SNP, single nucleotide polymorphism; BP, chromosome base pairs are based on human genome assembly version 19 (hg19); CA, coded allele; CAF, coded allele frequency; OR, odds ratio; SE, standard error of odds ratio; P_GC_, genomic controlled P-value.

Conditioning for the top SNP rs2187668 in males identified 12 SNPs at *P* < 5 × 10^–5^. The most significant SNP was rs9296079 (OR = 7.476, SE = 0.410, *P* = 9.51 × 10^–7^), located at 7.8 kb 5′ of *HLA-DPB2*. Similarly, conditioning for rs9268219 in females identified 8 SNPs at *P* < 5 × 10^–5^. The most significant was rs29243 (OR = 10.980, SE = 0.514, *P* = 6.04 × 10^–6^) located in *GABBR1*. Non-MHC SNPs at *P* < 5 × 10^–5^ were identified near *LRRTM4* in males and *CAST, LNPEP, TNIP1, CSMD1, B4GALNT1, SBNO2*, and *ABCG1* in females (Supplementary Table 3).

#### Non-LS sex groups

In the Swedish cohort (Supplementary Tables 4, 5), 5 SNPs in males at *P* < 3.5 × 10^–7^ and 1 SNP in females at *P* = 6.73 × 10^–7^ were identified. The top signals were rs1049550 (OR = 0.5231, SE = 0.1126, *P* = 1.82 × 10^–8^) located in *ANXA11* in males and rs1964995 (OR = 0.5314, SE = 0.1198, *P* = 6.73 × 10^–7^) located at 36 kb of the 3′ of *HLA-DRB5*, in females. After LD assessment, 3 SNPs in males defined as tag-SNPs remained.

In the German cohort (Supplementary Tables 6, 7), rs1964995 (OR = 0.5452, SE = 0.08223, *P* = 3.40 × 10^–11^) in males and rs4502931 (OR = 0.5899, SE = 0.06549, *P* = 4.36 × 10^–14^) in females were identified as the top signals. In the US African American cohort (Supplementary Tables 8, 9), rs9271640 (OR = 1.771, SE = 0.1097, *P* = 3.62 × 10^–7^) in males and rs1964995 (OR = 0.5452, SE = 0.08223, *P* = 3.40 × 10^–11^) in females were identified as the top signals.

The top association results for each cohort are summarized in Table 3. Manhattan and QQ plots for non-LS sex groups are shown in Supplementary Figures 2–4.

#### Interaction analysis with sex

Results from the interaction analysis in the LS and non-LS sex groups in the Swedish cohort showed significant SNPs interacting with the sex variable. In LS sex groups (Supplementary Table 10), the most significant interacting SNPs were rs2853973 (*P*_*int*_ = 3.8 × 10^–4^) in males and rs1470410 (*P*_*int*_ = 5.44 × 10^–4^) in females. In non-LS sex groups (Supplementary Table 11), the most significant interacting SNPs were rs10940422 (*P*_*int*_ = 4.18 × 10^–4^) in males and rs12432418 (*P*_*int*_ = 4.85 × 10^–5^) in females.

### Meta-analysis in non-LS

#### Non-LS sex groups

**FIGURE 2:**
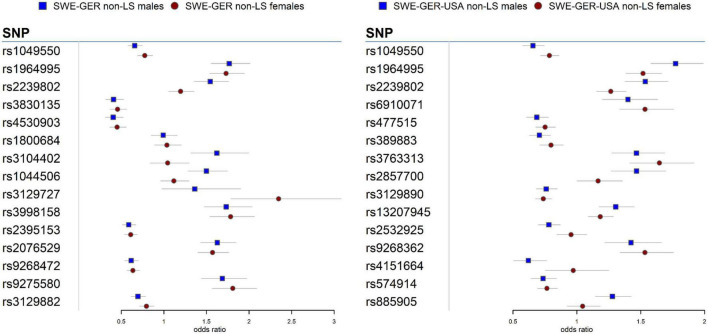
Forest plots for the 15 top common meta-SNPs in the non-LS sex groups in the European group (Swedish-German) and the multi-ethnic group (Swedish-German-United States-African American).

Meta-analysis in the European cohorts (Sweden and Germany) (Supplementary Tables 12, 13) identified 57 SNPs in males and 112 SNPs in females at *P*_*meta*_ < 5 × 10^–8^. Top signal rs1964995 located 36 kb from the 3′ of *HLA-DRB5* was the same in males (OR = 1.715, SE = 0.0656, *P*_*meta*_ = 3.92 × 10^–18^) and females (OR = 1.7304, SE = 0.0598, *P*_*meta*_ = 5 × 10^–20^). A forest plot illustrating the top findings is shown in Figure 2. Manhattan and Q-Q plots of the meta-analysis are shown in Supplementary Figure 5. Heterogeneity statistics in the SWE-GER are shown in Supplementary Table 13X.

**TABLE 3:** The top tag-SNPs (*P*_*GC*_ < 3.5 × 10^–7^) associated with non-LS males and females in Swedish, German, and USA-AA cohorts.

						Males				
SNP	CHR	BP (hg19)	Alleles	RefSeq genes	Cohort	CA	CAF	OR	95% CI	*P*_*GC*_
rs1049550	10	81,926,702	A/G	*ANXA11*	SWE	A	0.2962	0.5231	(0.4195, 0.6523)	1.82E-08
					GER	A	0.3845	0.7325	(0.6281, 0.8543)	3.63E-04
					USA-AA	A	0.1508	0.7724	(0.5841, 1.0215)	7.70E-02
rs2239802	6	32,411,846	C/G	*HLA-DRA*	SWE	C	0.3375	1.7790	(1.4295, 2.214)	4.52E-07
					GER	C	0.2283	1.4480	(1.2336, 1.6997)	4.75E-05
					USA-AA	C	0.3321	1.5140	(1.2288, 1.8655)	1.41E-04
rs7197	6	32,412,580	A/G	*HLA-DRA*	SWE	A	0.2885	1.7610	(1.4056, 2.2063)	1.50E-06
					GER	A	0.1901	1.4210	(1.1976, 1.6862)	2.96E-04
					USA-AA	A	0.2253	1.6350	(1.2895, 2.0731)	7.32E-05
rs1964995	6	32,449,411	G/A	*36kb 3′ of HLA-DRB5*	SWE	G	0.2995	0.6033	(0.4872, 0.747)	5.74E-06
					GER	G	0.3664	0.5452	(0.464, 0.6406)	3.40E-11
					USA-AA	G	0.2314	0.5568	(0.431, 0.7194)	1.21E-05
rs3830135	6	32,548,464	A/G	*HLA-DRB1*	SWE	A	0.0529	0.3543	(0.2278, 0.5512)	6.71E-06
					GER	A	0.0980	0.4374	(0.3212, 0.5956)	2.40E-06
					USA-AA	C	0.0702	0.5942	(0.3897, 0.906)	1.82E-02
rs3131283	6	32,119,898	A/G	*177bp 5′ of PRRT1*	SWE	A	0.1971	1.8100	(1.4054, 2.3312)	6.85E-06
					GER	A	0.1417	1.3790	(1.1382, 1.6707)	3.21E-03
					USA-AA	C	0.0256	1.7040	(0.9263, 3.1348)	9.41E-02
rs4530903	6	32,581,889	A/G	*23kb 5′ of HLA-DQA1*	SWE	A	0.0529	0.3590	(0.2308, 0.5585)	8.79E-06
					GER	A	0.0973	0.4282	(0.3133, 0.5853)	1.74E-06
					USA-AA	T	0.0702	0.5942	(0.3897, 0.906)	1.82E-02
rs1800684	6	32,151,994	T/A	*AGER*	SWE	T	0.1982	1.7920	(1.3917, 2.3076)	9.75E-06
					GER	A	0.1428	1.3810	(1.1401, 1.6729)	3.00E-03
					USA-AA	A	0.0293	1.6280	(0.9369, 2.8289)	9.13E-02
rs3104402	6	32,681,676	A/C	*27kb 5′ of HLA-DQA2*	SWE	A	0.1050	2.0860	(1.5013, 2.8984)	1.83E-05
					GER	T	0.0689	1.3750	(1.0539, 1.794)	3.50E-02
					USA-AA	G	0.0153	2.3790	(1.0553, 5.3629)	4.12E-02
rs1044506	6	32,172,065	A/C	*NOTCH4*	SWE	A	0.2016	1.7870	(1.3878, 2.3011)	1.08E-05
					GER	T	0.1441	1.3560	(1.1203, 1.6413)	4.97E-03
					USA-AA	G	0.0263	1.9640	(1.0695, 3.6067)	3.35E-02
						**Females**				
rs1964995	6	32,449,411	G/A	*36kb 3′ of HLA-DRB5*	SWE	G	0.2995	0.5314	(0.4202, 0.6721)	6.73E-07
					GER	G	0.3611	0.5942	(0.519, 0.6804)	1.65E-12
					USA-AA	G	0.2408	0.8051	(0.6963, 0.9309)	3.77E-03
rs3129727	6	32,679,690	A/G	*29kb 5′ of HLA-DQA2*	SWE	A	0.0474	3.0760	(2.0025, 4.7251)	1.37E-06
					GER	A	0.0268	1.9580	(1.3773, 2.7836)	4.53E-04
					USA-AA	T	0.0217	1.6270	(1.0686, 2.4773)	2.47E-02
rs3998158	6	32,681,992	G/A	*27kb 5′ of HLA-DQA2*	SWE	G	0.1700	0.5047	(0.3771, 0.6755)	1.49E-05
					GER	C	0.1990	0.5811	(0.4908, 0.6881)	3.67E-09
					USA-AA	C	0.1665	1.0890	(0.925, 1.2821)	3.10E-01
rs2395153	6	32,345,595	C/G	*5.9kb 5′ of C6orf10*	SWE	C	0.2748	0.5947	(0.4694, 0.7535)	5.05E-05
					GER	C	0.3391	0.6137	(0.5347, 0.7044)	7.74E-11
					USA-AA	G	0.2175	0.7410	(0.6372, 0.8618)	1.18E-04
rs2076529	6	32,363,955	G/A	*BTNL2*	SWE	G	0.3176	0.6105	(0.4859, 0.7672)	6.66E-05
					GER	C	0.3752	0.6458	(0.5659, 0.7371)	1.22E-09
					USA-AA	C	0.3056	0.8406	(0.7363, 0.9598)	1.11E-02
rs9268472	6	32,355,605	A/G	*6.9kb 3′ of BTNL2*	SWE	A	0.3176	0.6107	(0.486, 0.7674)	6.71E-05
					GER	T	0.3754	0.6446	(0.5648, 0.7357)	1.04E-09
					USA-AA	A	0.3063	0.8471	(0.7421, 0.967)	1.51E-02
rs9275580	6	32,679,462	G/A	*30kb 5′ of HLA-DQA2*	SWE	G	0.1791	0.5543	(0.4192, 0.7329)	9.63E-05
					GER	C	0.2102	0.5519	(0.4666, 0.6528)	7.77E-11
					USA-AA	G	0.2458	1.1700	(1.0138, 1.3503)	3.35E-02
rs3129882	6	32,409,530	G/A	*HLA-DRA*	SWE	G	0.5146	1.5580	(1.263, 1.922)	9.81E-05
					GER	G	0.4499	1.1700	(1.0356, 1.322)	1.85E-02
					USA-AA	A	0.4586	1.0660	(0.9446, 1.203)	3.05E-01

SNP, single nucleotide polymorphism; BP, chromosome base pairs are based on human genome assembly version 19 (hg19); CA, coded allele; CAF, coded allele frequency; OR, odds ratio; SE, standard error of odds ratio; P_GC_, genomic controlled P-value.

A comparison of associations at *P*_*meta*_ < 5 × 10^–8^ showed 44 SNPs shared between non-LS males and females, 17 SNPs exclusively associated with non-LS males, and 215 SNPs exclusively associated with non-LS females. Most SNPs were in the MHC region. Non-MHC SNPs include rs694739 (OR = 1.3626, SE = 0.063, *P*_*meta*_ = 9.21 × 10^–07^) located at 7.9 kbp from the 3′ of *PRDX5* associated with non-LS males, and rs2573346 (OR = 0.7712, SE = 0.0564, *P*_*meta*_ = 4.19 × 10^–06^) in *ANXA11* associated with non-LS females.

Results from a meta-analysis in the multi-ethnic cohorts (Sweden, Germany, and US-African American) (Supplementary Tables 14, 15) identified 12 SNPs in non-LS males and 49 in non-LS females (Table 3). Top SNPs were rs1964995 (OR = 1.7736, SE = 0.0587, *P*_*meta*_ = 1.54 × 10^–22^) located 36 kb from 3′ of *HLA-DRB5* in males, and rs2395153 (Beta = 0.6565, SE = 0.0477, *P*_*meta*_ = 1.11 × 10^–18^) located at 5.9 kb from 5′ of *C6orf10* in females. The top meta-SNPs are shown in Table 4. Forest plots (Figure 2) illustrate the top common meta-SNPs in the European and multi-ancestry cohort groups. Manhattan plots of meta-analysis in multi-ethnic groups are shown in Supplementary Figure 6. Heterogeneity statistics in SWE-GER-USA are shown in Supplementary Table 15X.

**TABLE 4:** The top significant SNPs (P_meta_ < 5e-8) of GWAS meta-analysis on non-LS sex groups.

Sex group (Cohorts)	SNP	CHR	BP	Alleles	RefSeq_ genes	CA	OR	95% CI	*P*-value	Direction	Het-I^2^	Het-PVal
Non-LS males (SWE-GER)	rs1964995	6	32,449,411	G/A	*36kb 3′ of HLA-DRB5*	A	1.7681	(1.5548, 2.0107)	3.92E-18	++	0	0.4583
	rs2395153	6	32,345,595	C/G	*5.9kb 5′ of C6orf10*	C	0.5861	(0.5134, 0.6692)	2.61E-15	–	0	0.5916
	rs2213585	6	32,413,150	G/A	*323bp 3′ of HLA-DRA*	A	0.6315	(0.5618, 0.71)	1.35E-14	–	0	0.3240
	rs7195	6	32,412,539	A/G	*HLA-DRA*	A	1.5830	(1.4082, 1.7795)	1.46E-14	++	14.5	0.2795
	rs9271588	6	32,590,953	G/A	*14kb 5′ of HLA-DQA1*	A	1.6153	(1.4291, 1.8258)	1.72E-14	++	0	0.5981
	rs2213586	6	32,413,094	A/G	*267bp 3′ of HLA-DRA*	A	1.5785	(1.4046, 1.7742)	1.93E-14	++	10	0.2918
	rs2227139	6	32,413,459	G/A	*632bp 3′ of HLA-DRA*	A	0.6335	(0.5637, 0.712)	1.93E-14	–	10	0.2918
	rs7192	6	32,411,646	A/C	*HLA-DRA*	A	1.5763	(1.4023, 1.7721)	2.54E-14	++	0.3	0.3165
	rs4373382	6	32,350,868	C/A	*11kb 5′ of C6orf10*	A	1.6291	(1.4337, 1.8512)	6.84E-14	++	0	0.6069
	rs2076529	6	32,363,955	G/A	*BTNL2*	A	1.6268	(1.4322, 1.8479)	7.03E-14	++	0	0.5625
Non-LS females (SWE-GER)	rs1964995	6	32,449,411	G/A	*36kb 3′ of HLA-DRB5*	A	1.7305	(1.5391, 1.9457)	5E-20	++	0	0.4192
	rs4502931	6	32,380,782	A/T	*5.9kb 5′ of BTNL2*	A	0.6033	(0.5401, 0.674)	3.82E-19	–	0	0.4968
	rs2395153	6	32,345,595	C/G	*5.9kb 5′ of C6orf10*	C	0.6088	(0.5405, 0.6859)	3.14E-16	–	0	0.8219
	rs9275582	6	32,680,070	A/G	*29kb 5′ of HLA-DQA2*	A	0.5499	(0.4757, 0.6358)	6.15E-16	–	0	0.7935
	rs2647012	6	32,664,458	A/G	*30kb 5′ of HLA-DQB1*	A	1.5502	(1.3899, 1.7291)	3.74E-15	++	0	0.5473
	rs9275393	6	32,669,439	A/G	*35kb 5′ of HLA-DQB1*	A	0.5890	(0.5156, 0.6728)	6.22E-15	–	0	0.3517
	rs2294878	6	32,367,795	A/C	*BTNL2*	A	0.6563	(0.5872, 0.7337)	1.27E-13	–	0	0.4173
	rs4530903	6	32,581,889	A/G	*23kb 5′ of HLA-DQA1*	A	0.4500	(0.364, 0.5564)	1.68E-13	–	0	0.5299
	rs2858332	6	32,68,1161	A/C	*28kb 5′ of HLA-DQA2*	A	0.6747	(0.6043, 0.7535)	2.83E-12	–	0	0.6205
	rs2858867	6	32,575,325	G/A	*18kb 5′ of HLA-DRB1*	A	0.6810	(0.6105, 0.7597)	5.54E-12	–	0	0.7201
Non-LS males (SWE-GER-USA-AA)	rs1964995	6	32,449,411	G/A	*36kb 3′ of HLA-DRB5*	A	1.7736	(1.5809, 1.9899)	1.54E-22	+++	0	0.7552
	rs2239802	6	32,411,846	C/G	*HLA-DRA*	C	1.5360	(1.3764, 1.7143)	1.88E-14	+++	19.6	0.2883
	rs4530903	6	32,581,889	A/G	*23kb 5′ of HLA-DQA1*	A	0.4478	(0.36, 0.5571)	5.40E-13	—	27.7	0.2506
	rs477515	6	32,569, 691	A/G	*12kb 5′ of HLA-DRB1*	A	0.6866	(0.607, 0.7767)	2.21E-09	—	0	0.4141
	rs389883	6	31,947,460	C/A	*STK19*	A	0.7073	(0.6302, 0.7939)	4.02E-09	—	0	0.3934
	rs3763313	6	32,376,471	C/A	*1.6kb 5′ of BTNL2*	A	1.4667	(1.2747, 1.6877)	9.03E-08	+++	21.6	0.2794
	rs9268835	6	32,428,115	A/G	*15kb 3′ of HLA-DRA*	A	0.7131	(0.6293, 0.8081)	1.13E-07	—	0	0.6909
	rs2857700	6	31,572,481	A/G	*11kb 5′ of AIF1*	A	1.4676	(1.27, 1.696)	2.02E-07	+++	16.1	0.3036
	rs3129890	6	32,414,273	G/A	*1.4kb 3′ of HLA-DRA*	A	0.7603	(0.6831, 0.8464)	5.58E-07	—	64.4	0.0604
	rs2269425	6	32,123,639	A/G	*PPT2*	A	0.6415	(0.538, 0.765)	7.69E-07	—	0	0.9529
Non-LS females (SWE-GER-USA-AA)	rs2395153	6	32,345,595	C/G	*5.9kb 5′ of C6orf10*	C	0.6565	(0.598, 0.7209)	1.11E-18	—	50.8	0.1311
		rs7195	6	32,412,539	A/G	*HLA-DRA*	A	1.4402	(1.3254, 1.5651)	7.42E-18	+++	0	0.8684
		rs2294878	6	32,367,795	A/C	*BTNL2*	A	0.7053	(0.6471, 0.7689)	2.06E-15	—	57.1	0.0971
		rs3129890	6	32,414,273	G/A	*1.4kb 3′ of HLA-DRA*	A	0.7374	(0.6772, 0.8031)	2.61E-12	—	20.5	0.2843
		rs6913309	6	32,339,840	A/T	*183bp 5′ of C6orf10*	A	0.7060	(0.6397, 0.7792)	4.25E-12	—	0	0.5575
		rs2269425	6	32,123,639	A/G	*PPT2*	A	0.6160	(0.5359, 0.7082)	9.74E-12	—	5.5	0.3471
		rs3129727	6	32,679,690	A/G	*29kb 5′ of HLA-DQA2*	A	2.1069	(1.6768, 2.6473)	1.62E-10	+++	56.6	0.1000
		rs6910071	6	32,282,854	G/A	*C6orf10*	A	1.5340	(1.3392, 1.7573)	6.61E-10	+++	0	0.4492
		rs3115572	6	32,220,484	C/G	*29kb 5′ of NOTCH4*	C	1.2871	(1.1875, 1.3951)	8.48E-10	+++	14.3	0.3113
		rs3817963	6	32,368,087	G/A	*BTNL2*	A	1.3835	(1.2453, 1.5371)	1.53E-09	+++	0	0.8790

SNP, single nucleotide polymorphism; CHR, chromosome; BP, base pairs; hg19, human genome assembly version 19; OR, odds ratio; 95% CI, 95% confidence interval; P_meta_, metal P-value; Direction: (–) reversed strand; (++) forward strand; Het-I^2^, heterogeneity I-squared; Het-PVal, heterogeneity I-squared P-value.

A comparison analysis at *P*_*meta*_ < 5 × 10^–8^ showed a shared SNP between non-LS males and females, 11 SNPs exclusively associated with non-LS males, and 48 SNPs exclusively associated with non-LS females. Most associated SNPs were in the MHC region. Non-MHC SNPs were observed in non-LS females and included rs7813186, rs1049550, and rs7133604 (Data not shown).

### Lookup of meta-SNPs in the UK Biobank

Genome-wide association study of “Doctor diagnosed sarcoidosis” in the UKB was used as a validation step. SNP-lookup showed several sex-specific SNPs in LS and non-LS at *P* < 5 × 10^–5^. Chiefly, 223 SNPs associated with LS males and 209 SNPs associated with LS females were validated at *P* < 5 × 10^–8^ (Supplementary Tables 16, 17). In non-LS, 11 non-LS males and 31 SNPs non-LS females in the SWE-GER cohorts were validated at *P* < 5 × 10^–8^ (Supplementary Tables 18, 19). In the multi-ethnic cohorts (SWE-GER-USA-AA), 2 SNPs in non-LS males and 8 SNPs in non-LS females were observed (Supplementary Tables 20, 21).

### Gene-based analysis

The gene-based analysis revealed significant genomic loci associated with sex groups in LS and non-LS (Supplementary Tables 22, 23). The most significant genomic locus was *HLA-DRA* (*P* = 4.36 × 10^–14^) in LS males and *FKBPL* (*P* = 1.39 × 10^–14^) in LS females at a gene-based *P* < 2.0 × 10^–6^ (0.05/24,769 genes).

In the European cohorts (SWE-GER), 15 genes associated with non-LS males and 18 genes associated with non-LS females were identified, where the most significant genomic locus was *HLA-DRA* (*P* = 5.16 × 10^–15^) in non-LS males and *HLA-DRB1* (*P* = 2.42 × 10^–13^) in non-LS females. In the multi-ethnic cohorts (SWE-GER-USA-AA), 5 genes associated with non-LS males and 8 genes associated with non-LS females were identified. The top genes were *HLA-DRA* (*P* = 1.88 × 10^–14^) in non-LS males and *C6orf10* (*P* = 6.47 × 10^–12^) in non-LS females.

A comparison of gene-based analysis in LS and non-LS sex groups across cohorts (Supplementary Table 24) revealed differences in gene-based associations. In LS, gene-based associations showed 77 genes shared among sex groups, 11 genes exclusively associated with males, and 14 genes exclusively associated with females. In non-LS sex groups, in the European cohorts (SWE-GER), 18 genes were shared among non-LS sex groups, and 8 genes were exclusively associated with non-LS males. In the multi-ethnic cohorts (SWE-GER-USA-AA), 4 genes were shared, one gene was exclusively associated with non-LS males, and 4 were exclusively associated with non-LS females.

**FIGURE 3:**
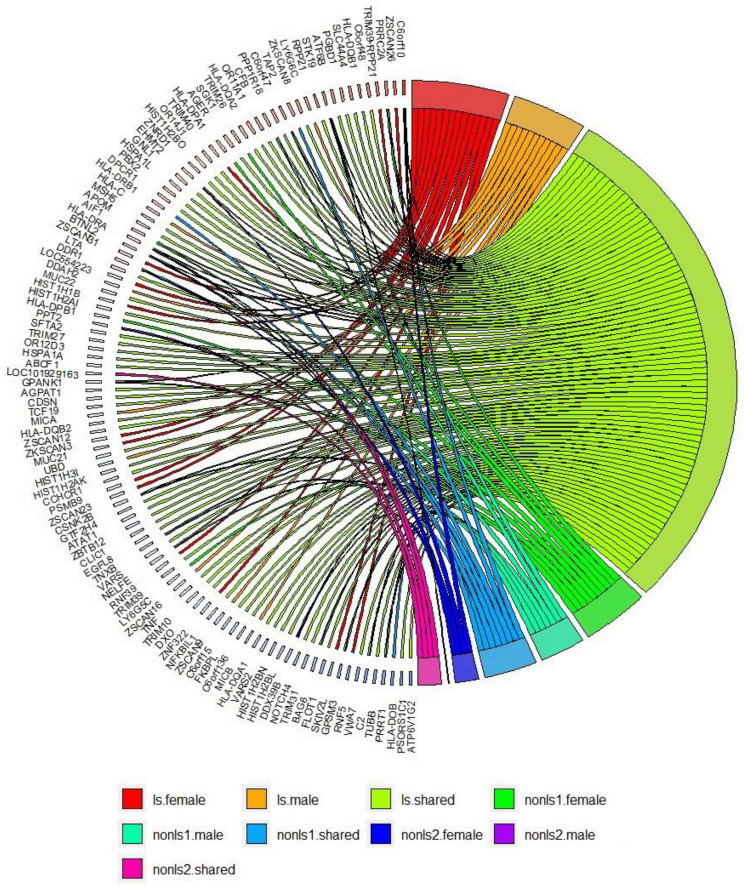
Gene-based analysis in LS and non-LS sex groups across all cohorts at *p* < 2e-6. ls, LS in SWE; non-ls1, non-LS in SWE and GER; non-ls2, non-LS in SWE, GER, and USA-AA; shared, genes shared by sex groups; male, genes associated with males only; female, genes associated with females only.

Figure 3 shows a circos plot depicting gene-based associations among LS and non-LS sex groups across cohorts.

### Gene expression and expression quantitative trait loci (eQTL) enrichment

Results from tissue expression analysis using 54 tissue types in GText version 8 and FUMA revealed significant sex-specific gene expression at Bonferroni *P*-value (*P*_*Bon*_ < 0.05).

Löfgren’s syndrome males and females (Supplementary Figure 7), significant differential gene expression was observed in the spleen, small intestine, lung, and blood.

In the European cohorts (SWE, GER) (Supplementary Figure 8), differential gene expression was observed in the spleen, small intestine terminal Ileum, lung, and blood in non-LS males. In contrast, significant differential gene expression was observed in the brain hippocampus, and lung in non-LS females. In the multi-ethnic cohorts (SWE, GER, USA-AA) (Supplementary Figure 8), differentially expressed genes were observed in the spleen, small intestine terminal ilium, lung, and blood in non-LS males and females.

Expression quantitative trait loci enrichment using different eQTL databases across different tissues and cell types (Supplementary Table 25) revealed several significant eQTLs SNPs (eSNPs) in sex groups.

In LS sex groups, 311 eSNPs in 134 genes in males and 354 eSNPs in 140 genes in females were identified using the GText v8 database. In SWE-GER cohorts, non-LS sex groups, 32 eSNPs in 77 genes in males and 59 eSNPs in 89 genes in females were identified using the GText v8 database. In the SWE-GER-US-AA cohorts, non-LS sex groups, two eSNPs in males and 23 eSNPs in females were identified using the GText v8 database. Similar observations were also observed using eQTLGen and eQTLcatalogue databases.

Moreover, eQTL mapping using immune cell eQTLs (Supplementary Table 26) available in the EUGENE database identified significant eSNPs in relevant immune cell types. In LS sex, significant eSNPs of B cells, T cells, CD4, and CD8 T cells, granulocytes, monocytes, macrophages, neutrophils, and PBMCs were identified. In the SWE-GER-USA-AA non-LS sex groups, eQNPS of T cells, CD4 T cells, fibroblasts, monocytes, macrophages, and neutrophils were observed.

### Gene enrichment and pathway analysis

In LS sex groups (Supplementary Table 27), a pathway map related to immune response induction of the antigen presentation machinery by IFN-gamma (FDR = 1.530e-8 in males; FDR = 1.926e-11 in females) were identified. In the SWE-GER non-LS sex groups (Supplementary Table 27), pathway maps related to maturation and migration of dendritic cells in skin sensitization (FDR = 2.085e-9) in males and related to immune response induction of the antigen presentation machinery by IFN-gamma (FDR = 1.998e-10) in females were identified. In the SWE-GER-USA-AA non-LS sex groups (Supplementary Table 27), pathway maps related to immune response lectin-induced complement pathway (FDR = 1.571e-5) in males and related to maturation and migration of dendritic cells in skin sensitization (FDR = 1.824e-6) in females were noted.

## Discussion

The present study investigated sex differences in sarcoidosis, particularly in LS and non-LS phenotypes. This work is the first investigation for independently characterizing the genetic architecture of sarcoidosis in males and females. In brief, we analyzed genetic associations between LS and non-LS males and females conducted in European and multi-ethnic cohorts. The multi-ethnic cohorts comprised three population-based cohorts from Sweden, Germany, and the United States. The cohorts included different ancestries, i.e., white European and black African American.

For both LS and non-LS sex groups, a genetic analysis based on a genome-wide association study was conducted, followed by a meta-analysis, an SNP lookup in the UKB, gene-based analysis, differential gene expression analysis using the GText version 8 resource, eQTL tissue/cell mapping enrichment, and pathway analysis.

Löfgren’s syndrome, an acute form of sarcoidosis, is highly prevalent in Sweden (55). However, due to insufficient LS cases in the German cohort and being rare in the United States, genetic analyses in the LS sex groups were only conducted in the Swedish cohort. In contrast, genetic investigations in the non-LS sex groups were performed across all three cohorts.

Association analysis in LS sex groups identified distinct genetic associations clustering in the extended MHC (xMHC) (56), highlighting the role of genes located in this region as main component of LS susceptibility, as previously reported (23). The top LS findings were associations in *HLA-DQA1* in males and *C6orf10* in females. Although these genomic loci are on the same chromosome region (classical class II) and many variants in these genes are in linkage disequilibrium (LD *r*^2^ > 0.8), we observed significant differences in the genetic effects (beta estimates) among variants associated with LS males and females (Figure 1). Interestingly, after conditioning for the top MHC signals, we identified non-MHC loci worth investigating in future studies of LS. For instance, a nearby signal close to *LRRTM4* associated with LS males is worth looking into, as *LRRTM4* is a synapse-organizing molecule involved in protein-protein interactions that regulate the nervous and immune systems (57). Another interesting non-MHC association is *LNPEP* associated with LS females. *LNPEP* is an aminopeptidase that regulates hormones (i.e., arginine-vasopressin and oxytocin), and is involved in trimming peptide antigens for cross-presentation to T cells in autoimmune diseases (58).

In the European cohorts (SWE and GER), meta-analysis in non-LS sex groups identified mainly associations in the MHC classical class II subregion and a few non-MHC signals, chiefly in *ANXA11, TMEM163*, and nearby *PRDX5* in both sex groups. As in LS, the differences among these signals were the genetic effects (beta estimates) in the sex groups, as illustrated in the forest plot (Figure 2). Similar findings were observed in the multi-ethnic cohorts (SWE, GER, and USA-AA).

To further understand the genetic differences, the comparison of genetic signals of LS and non-LS sex groups showed that some signals were associated with a specific sex, whereas other signals were common to both males and females; however, their genetic effects differed. These observations were then validated using the UKB resource, using the “doctors diagnosed sarcoidosis” phenotype. Based on this evidence, it is compelling to suggest that genetic differences in the sex groups could be linked to the observed differences in clinical manifestations in sarcoidosis male and female patients, as documented in various works (59–61). Additionally, it is essential to highlight that ancestry also played a role in the differences in genetic effects in the sex groups, as the effects changed when multi-ethnic ancestries were considered.

Results from gene-based analysis showed patterns of genomic associations in the sex groups, such as shared and sex-specific (Figure 3). Notably, the genomic patterns of associations in the LS sex groups were more extensive since the LS susceptibility spanned across the xMHC, which harbors 252 genes and 139 pseudogenes. In the non-LS sex groups, on the contrary, the genomic patterns were limited to a few genes located in the MHC class II region. As expected, genomic patterns decreased as more ancestries were included, stressing the role of ancestry as a modifier of sarcoidosis susceptibility. Ancestry plays a significant role in the genetics of complex diseases (62, 63), which is also relevant in sarcoidosis.

Insights from tissue gene expression and eQTL enrichment showed that SNPs associated with biological sex play a role in gene expression variability across various tissues and cell types. For instance, gene expression in the spleen, small intestine, lung, and whole blood differed in LS and non-LS sex groups. Furthermore, SNP associations correlated with eQTLs of tissues and cell types, particularly immune cells eQTLs (e.g., B cells, T cells, CD4 T cells, CD8 T cells, macrophages, and monocytes), were identified. These findings underscore the functional role of genetic variants on gene expression of immune cells, thus shaping the immunopathogenesis of sarcoidosis in the sex groups. eQTL studies in complex diseases showed that eQTL SNPs act as master regulators of gene expression in several tissues and cell types (64, 65), influencing the expression of multiple genes and acting as gene regulators (66).

Expression quantitative trait loci findings in various immune cell types (Supplementary Table 26) further supports our hypothesis that sarcoidosis is an immune-regulated disease, as previously proposed (67–69).

Indeed, differences in SNP associations in HLA genes (70–75) may result in different immune responses and inflammatory disease course as seen in male and female patients with sarcoidosis. Thus, it is worth keeping in mind that HLA genes play a crucial role in autoimmunity and that their function has a profound sex bias on disease phenotype, as more females are affected by autoimmune (or chronic inflammatory) disorders than the counterparts. Understanding the molecular mechanisms underlying sex selection in the development of autoimmune disorders is an area of active research (76).

Compiling the evidence shown in this work, with a particular focus on the strong presence of HLA genetic associations in sarcoidosis, the involvement of various immune cells types, and the differences in immune response and clinical course in sex groups (67, 68, 77), the possibility of sarcoidosis being an autoimmune disorder cannot be ignored.

Besides HLA genes, we also identified signals in the *ANXA11* gene in non-LS sex groups with different genetic effects, as denoted by rs1049550 (Figure 2). SNP associations in *ANXA11* were first reported by Hofmann et al. (78) and have been linked to sarcoidosis risk, radiographic phenotype (Scadding stage IV), and to interact with HLA-DRA (79). SNPs in *ANXA11* have also been reported to be associated with autoimmune diseases (80). Indeed, the functional role of *ANXA11* in cell division and apoptosis (81) and the regulation of inflammatory cells (82) make *ANXA11* an attractive biomarker for sarcoidosis.

Moreover, evidence for a pathway related to immune response induction of the antigen presentation machinery by IFN-gamma in LS sex groups offers an incentive to examine the role of IFN-gamma in sarcoidosis (Supplementary Table 27). IFN-gamma is a pro-inflammatory cytokine produced by immune cells and has immune effector functions on many genes involved in tissue homeostasis, immune/inflammatory responses, and tumor immunosurveillance (83). In the non-LS groups, pathway maps identified mainly were related to immune response but not specific to a sex group. Thus, more data is needed to understand the underlying mechanisms of non-LS as this sarcoidosis phenotype is heterogeneous and polygenic.

In summary, we provide new insights into a sex bias underpinning the genetic architecture of sarcoidosis. The differences observed in the genetic susceptibility in the sex groups in LS and non-LS add further information which may explain the well-recognized observations between sex groups, incidence, prevalence, age of onset, symptoms, severity of the disease, and drug reactions observed in male and female sarcoidosis patients (23, 84–87).

It goes without a doubt that further genetic and omics studies ought to be conducted to broaden the characterization of the genetic architecture of sarcoidosis among sex groups. Such efforts, of course, shall consider the inclusion of multi-ancestry populations with large sample sizes and balanced sex ratios. An ongoing endeavor to meet such requirements is the MESARGEN consortium,^2^ an international framework aimed to include multiple ancestry populations and large sample sizes for genetics and genomics studies of sarcoidosis.

### Limitations of the study

We used the Illumina Immunochip version 2 platform in our analysis—a customized design based on investigating major autoimmune and inflammatory diseases (88). Therefore, we limited our investigations to immune-mediated genomic regions, leaving out other potential loci implicated in sarcoidosis susceptibility. Despite this limitation, we offer substantial evidence that the genetic susceptibility of sarcoidosis and LS and non-LS phenotypes in males and females shared common genomic loci but also differed in their genetic architectures given the sex group. We observed these differences in gene-based analysis, eQTLs enrichment across different tissues, and pathway analysis. Further investigations with multiple ancestry populations and large sample sizes are needed to unveil discoveries.

### Perspectives and significance

In this work, we revealed sex differences in the genetic architecture of sarcoidosis, particularly in clinical phenotypes LS and non-LS. Our work provides further knowledge that the genetic architectures of LS and non-LS are distinct, and that biological sex plays a role in determining the underlying genetic architecture.

## Data availability statement

Access to genomic data is limited due to GPDR restrictions because the data contain personal or other sensitive information and cannot be deposited in public databases. All summary statistics from the analyses are accessible via DOI: 10.6084/m9.figshare.21785273.

## Ethics statement

This study was approved by the Stockholm Ethical Review Board, Stockholm, Sweden. The patients/participants provided their written informed consent to participate in this study.

## Author contributions

NR and LP designed and implemented the study. YX conducted all the genetic analyses in the Swedish cohort and a meta-analysis among all cohorts. YX and NR drafted the manuscript. SK, JG, and AE recruited patients, obtained samples, and clinically evaluated patients for phenotype characterization in the Swedish cohort. DE conducted genetic analyses in the German cohort. SS and JM-Q recruited patients, obtained samples, and clinically evaluated patients for phenotype characterization in the German cohort. BR and MI recruited patients and obtained samples. CM provided genotype data and oversaw analyses conducted by LG and NP of the US-AA cohort. All authors revised the manuscript and provided feedback.

## Footnotes

1
http://prioritypruner.sourceforge.net/

2
http://mesargen.org
