# Corrigendum: Sex differences in the genetics of sarcoidosis across European and African ancestry populations

**DOI:** 10.3389/fmed.2024.1382584

**Published:** 2024-02-21

**Authors:** Ying Xiong, Susanna Kullberg, Lori Garman, Nathan Pezant, David Ellinghaus, Vasiliki Vasila, Anders Eklund, Benjamin A. Rybicki, Michael C. Iannuzzi, Stefan Schreiber, Joachim Müller-Quernheim, Courtney G. Montgomery, Johan Grunewald, Leonid Padyukov, Natalia V. Rivera

**Affiliations:** ^1^Respiratory Medicine Division, Department of Medicine Solna, Karolinska Institutet, Stockholm, Sweden; ^2^Department of Respiratory Medicine and Allergy, Theme Inflammation and Ageing, Karolinska University Hospital, Stockholm, Sweden; ^3^Genes and Human Disease, Oklahoma Medical Research Foundation, Oklahoma City, OK, United States; ^4^Institute of Clinical Molecular Biology, Christian-Albrechts-University of Kiel, Kiel, Germany; ^5^Department of Public Health Sciences, Henry Ford Health System, Detroit, MI, United States; ^6^Zucker School of Medicine, Staten Island University Hospital, Northwell/Hofstra University, Staten Island, NY, United States; ^7^Clinic for Internal Medicine I, University Hospital Schleswig-Holstein, Kiel, Germany; ^8^Department of Pneumology, University Medical Center Freiburg, Faculty of Medicine, University of Freiburg, Freiburg im Breisgau, Germany; ^9^Center for Molecular Medicine, Karolinska Institutet, Stockholm, Sweden; ^10^Division of Rheumatology, Department of Medicine Solna, Karolinska Institutet, Karolinska University Hospital, Stockholm, Sweden

**Keywords:** sarcoidosis, genetics, genome-wide association study (GWAS), meta-analysis, single nucleotide polymorphisms, immunogenetics and HLA

In the published article, there was an error in the Funding statement. The source and funding of the Genome Sequencing data of the African-American participants were inaccurately reported.

The correct Funding statement appears below.

