# Supplementary material for: Sex differences in the genetics of sarcoidosis across European and African ancestry populations
Source: Front Med (Lausanne). 2023 May 11;10:1132799. doi: 10.3389/fmed.2023.1132799 (PMC10213734; doi:10.3389/fmed.2023.1132799)
Supplement: Supplementary file 1 [file Data_Sheet_1.pdf]

Sex differences in the genetics of sarcoidosis across European and African ancestry populations

Short title: Sex differences in the genetic architecture of sarcoidosis

Ying Xiong[1], Susanna Kullberg[1,2], Lori Garman[3], Nathan Pezant[3], David Ellinghaus[4], Vasiliki Vasila[1], Anders Eklund[2], Benjamin A. Rybicki[5], Michael C. Iannuzzi[6], Stefan Schreiber[4,7], Joachim Müller-Quernheim[8], Courtney G. Montgomery[3], Johan Grunewald[1,2,10] Leonid Padyukov[9,10], and Natalia V. Rivera[1,9,10*]

[1]Respiratory Medicine Division, Department of Medicine Solna, Karolinska Institutet, Stockholm, Sweden

[2]Department of Respiratory Medicine and Allergy, Theme Inflammation and Ageing, Karolinska University Hospital, Stockholm, Sweden,

[3]Genes and Human Disease, Oklahoma Medical Research Foundation, Oklahoma City, OK, USA

[4]Institute of Clinical Molecular Biology, Christian-Albrechts-University of Kiel, Kiel, Germany

[5]Department of Public Health Sciences, Henry Ford Health System, Detroit, MI, USA

[6]Staten Island University Hospital, Zucker School of Medicine, Northwell/Hofstra University, Staten Island, NY, USA

[7]Clinic of Internal Medicine I, University Hospital Schleswig-Holstein, Campus Kiel, Kiel, Germany

[8] Department of Pneumology, Medical Center, University of Freiburg, Faculty of Medicine, University of Freiburg, Germany

[9]Center for Molecular Medicine, Karolinska Institutet, Stockholm, Sweden

[10]Division of Rheumatology, Department of Medicine Solna, Karolinska Institutet and Karolinska University Hospital, Stockholm, Sweden

***Corresponding author**

Natalia V Rivera, PhD

Respiratory Medicine Division, Department of Medicine Solna, Karolinska Institutet, Stockholm, Sweden

Email: natalia.rivera@ki.se

Tables and Figures in Supplementary Materials are available at figshare DOI: 10.6084/m9.figshare.21785273

<u>Figures</u>

Supplementary Figure S1. Manhattan and Q-Q plots for LS males and females in the Swedish cohort

Supplementary Figure S2. Manhattan and Q-Q plots for non-LS males and females in the Swedish cohort

Supplementary Figure S3. Manhattan and Q-Q plots for non-LS males and females in the German cohort

Supplementary Figure S4. Manhattan and Q-Q plots for non-LS males and females in the USA-AA cohort

Supplementary Figure S5. Manhattan and Q-Q plots of Meta-analysis of non-LS sex groups in Swedish and German cohorts

Supplementary Figure S6. Manhattan and Q-Q plots of Meta-analysis of non-LS sex groups in Swedish, German, and USA-AA cohorts

Supplementary Figure S7. Differentially gene expression in LS sex groups in the Swedish cohort

Supplementary Figure S8. Differentially gene expression in non-LS sex groups across different cohorts

<u>Tables</u>

Supplementary Table S1. Results from SNP association analysis in LS males in the Swedish cohort

Supplementary Table S2. Results from association analysis in LS females in Swedish cohort

Supplementary Table S2X. Summary of heritability statistics for LS sex groups (SWE)

Supplementary Table S3. Conditional analysis of top SNP in LS sex groups

Supplementary Table S4. Results from association analysis in non-LS males in Swedish cohort

Supplementary Table S5. Results from association analysis in non-LS females in Swedish cohort

Supplementary Table S6. Results from association analysis in non-LS males in German cohort

Supplementary Table S7. Results from association analysis in non-LS females in German cohort

Supplementary Table S8. Results from association analysis in non-LS males in USA-AA

Supplementary Table S9. Results from association analysis in non-LS females in USA-AA

Supplementary Table S10. Interaction analysis in LS sex groups in the Swedish cohort

Supplementary Table S11. Interaction analysis in non-LS sex groups in the Swedish cohort

Supplementary Table S12. Results from meta-analysis of non-LS males in Swedish and German cohorts

Supplementary Table S13. Results from meta-analysis non-LS females in Swedish and German cohorts

Supplementary Table S14. Results from meta-analysis non-LS males in multi-ethnic cohorts (Sweden, Germany, and USA-AA)

Supplementary Table S15. Results from meta-analysis non-LS females in multi-ethnic cohorts (Sweden, Germany, and USA-AA)

Supplementary Table S16. UKBB SNP look-up in LS males in the Swedish cohort

Supplementary Table S17. UKBB SNP look-up in LS females in the Swedish cohort

Supplementary Table S18. UKBB SNP look-up in non-LS males in the Swedish and German cohorts

Supplementary Table S19. UKBB SNP look-up in non-LS females in the Swedish and German cohorts

Supplementary Table S20. UKBB SNP look-up in non-LS males in the Swedish, German, and USA-AA cohorts

Supplementary Table S21. UKBB SNP look-up in non-LS females in the Swedish, German, and USA-AA cohorts

Supplementary Table S22. Results from gene-base analysis on LS sex groups in the Swedish cohort at P< 2e-6

Supplementary Table S23. Results from gene-base analysis on non-LS sex groups in SWE-GER and SWE-GER-USA-AA cohorts at P< 2e-6

Supplementary Table S24. Results from gene-based comparison analysis in LS and non-LS sex groups across cohorts at P< 2e-6

Supplementary Table S25. Summary of eQTL mapping in LS and non-LS sex groups across cohorts in different eQTL databases

Supplementary Table S26. Summary of eQTL immune cell mapping with Eugene database in LS and non-LS sex groups
